# High fat diet exacerbates Alzheimer's disease-related pathology in APPswe/PS1 mice

**DOI:** 10.18632/oncotarget.12179

**Published:** 2016-09-21

**Authors:** Peter Thériault, Ayman ElAli, Serge Rivest

**Affiliations:** ^1^ Neuroscience Laboratory, CHU de Québec Research Center and Department of Molecular Medicine, Faculty of Medicine, Laval University, Québec City, QC, Canada; ^2^ Neuroscience Laboratory, CHU de Québec Research Center and Department of Psychiatry and Neuroscience, Faculty of Medicine, Laval University, Québec City, QC, Canada

**Keywords:** Alzheimer's disease, age, high fat diet, blood-brain barrier, pericyte, Pathology Section

## Abstract

Alzheimer's disease (AD) is mainly characterized by the accumulation and aggregation of amyloid-β (Aβ) peptides in brain parenchyma and cerebral microvasculature. Unfortunately, the exact causes of the disease are still unclear. However, blood-brain barrier (BBB) dysfunction and activation of inflammatory pathways are implicated in AD pathogenesis. Importantly, advanced age and high fat diet, two major risk factors associated with AD, were shown to deeply affect BBB function and modulate the immune response. As such, this study evaluated the impact of age and high fat diet on AD progression. For this purpose, 3 (i.e. young) and 12 (i.e. aged) months old APPswe/PS1 mice were fed for 4 months with a high fat diet (i.e. Western diet (WD)) or normal diet. Interestingly, neurobehavioral tests revealed that WD accelerates age-associated cognitive decline without affecting parenchymal Aβ. Nonetheless, WD decreases matrix metalloproteinase-9 enzymatic activity and brain-derived neurotrophic factor mRNA and protein levels in brain, suggesting loss of synaptic plasticity. In the periphery, WD promotes systemic inflammation by increasing the levels of blood-circulating monocytes and monocyte chemotactic protein-1 production, which is accompanied by an augmentation of oxidized-low density lipoprotein levels in blood circulation. At the BBB, WD potentiates the age-induced increase of Aβ 1-40 accumulation and exacerbates the oxidative stress, specifically in cerebral microvasculature. These effects were accompanied by the dysfunction of pericytes, thus altering BBB functionality without compromising its integrity. Our study provides new insights into the implication of high fat diet in accelerating the cognitive decline in AD.

## INTRODUCTION

Alzheimer's disease (AD) is the most prevalent neurodegenerative disease in elderly population worldwide. AD patients display a progressive loss of memory and cognitive deficits that evolve over time from mild to severe cognitive decline, and ultimately, to the loss of executive functions [1]. The main pathological hallmarks of AD are amyloid-β (Aβ) deposition, constituted by Aβ 1-40 and Aβ 1-42 peptides, and neurofibrillary tangle formation in the brain [2]. It is now well recognized that soluble oligomeric Aβ species play a neurotoxic role, since their formation and accumulation within the brain correlates with neuronal loss and cognitive decline [1, 2]. Although several risk factors have been linked to an increased prevalence for AD development, the exact causes of this disease are still elusive.

In late-onset form of AD, advanced age constitutes the main risk factor associated to AD [1]. Age-related factors affect several key functions, such as cell senescence, mitochondrial dysfunction, altered intercellular communication and stem cell exhaustion [3]. These factors deeply alter the functionality of various biological systems including the brain that rely on synaptic plasticity, a physiological process that is essential for dendrite remodeling depending on neuronal activity, which is controlled by several sophisticated mechanisms among which are enzymatic activity of matrix metalloproteinase-9 (MMP-9) [4, 5] and brain-derived neurotrophic factor (BDNF) gene expression, a long-term potentiation (LTP)- related gene [6], within brain parenchyma. Besides, aging alters the function of cerebral microvasculature [7] and innate immune system [8]. Monocytes are blood -circulating mononuclear phagocytes, comprising a pro-inflammatory (Ly6C^High^) and patrolling (Ly6C^Low^) subset, which are both involved in debris phagocytosis and secretion of soluble mediators namely monocyte chemotactic protein-1 (MCP-1) [9]. Aging contributes to a phenomenon called “inflamm-aging”, which is an age-induced deregulation of the innate immune system that promotes chronic systemic inflammation [3]. More precisely, senescent immune cells secrete higher levels of inflammatory molecules in the blood circulation [10], such as reactive oxygen species (ROS) that contribute to oxidative stress [11, 12]. Importantly, it has been reported that excessive monocytosis coincides with systemic inflammation [13, 14].

Several studies have thoroughly tried to identify genetic (e.g. apolipoprotein E4, ApoE4) [15] and environmental (e.g. diabetes) risk factors [16, 17] involved in AD pathogenesis. Poor diets, including high fat diets, have been recognized as major risk factor for chronic disorders, namely diabetes, hypertension and obesity, thus increasing by 10% to 25% the risk of developing AD [16, 18]. High fat diets contain usually high levels of simple carbohydrates, saturated fatty acids and cholesterols, thus inducing metabolic disturbances such as hyperglycemia and hyperlipidemia [19, 20]. High fat diets have been shown to increase the levels of low-density lipoproteins (LDL) in plasma, which is highly susceptible to be oxidized, leading to the generation of oxidized-LDL (ox-LDL), a highly reactive molecule that has been associated to systemic inflammation and vascular damage, such as atherosclerosis [21-23]. In this regard, previous studies have outlined the early contribution of cerebral microvasculature dysfunction in AD development [24, 25].

Cerebral microvasculature is constituted by the blood-brain barrier (BBB), which is a highly dynamic structure formed by tightly sealed endothelial cells, *via* tight junction proteins (e.g. claudin-5, occludin) that delimitate two functionally distinct sides, the luminal side facing blood circulation and the abluminal side facing brain parenchyma [7]. The BBB has two major properties, physical associated to permeability, and functional associated to transport, regulating the exchange between the periphery and brain parenchyma [7]. In fact, the BBB precisely controls brain homeostasis by maintaining the delivery of oxygen and nutrients into the brain, and eliminating toxic metabolites from brain parenchyma through various transporters including ATP-binding cassette sub-family B member 1 (ABCB1) [7, 26]. As such, the BBB tightly cooperates with periphery and brain parenchyma in order to eliminate Aβ species from the brain [7]. However, the impaired clearance of Aβ species across the BBB has been proposed to contribute to the development of cerebral amyloid angiopathy (CAA), which takes place in 80% of AD cases [27]. At the abluminal side, BBB function is controlled by pericytes [7, 28]. Interestingly, the degeneration or dysfunction of pericytes has been observed in post-mortem tissues of AD patients [29, 30] and studied in AD animal models [31], thus suggesting their implication in AD pathogenesis. Nevertheless, little is known about the impact of high fat diet on pericyte function, and ultimately, in AD development.

In this study, we aimed to investigate the synergistic role of age and high fat diet in AD progression. Our findings unravel new insights in the implication of high fat diet in exacerbating AD pathogenesis and progression, mainly by affecting cerebral microvasculature function.

## RESULTS

### WD increases body weights and exacerbates cognitive deficits of APPswe/PS1 mice

**Figure 1:**
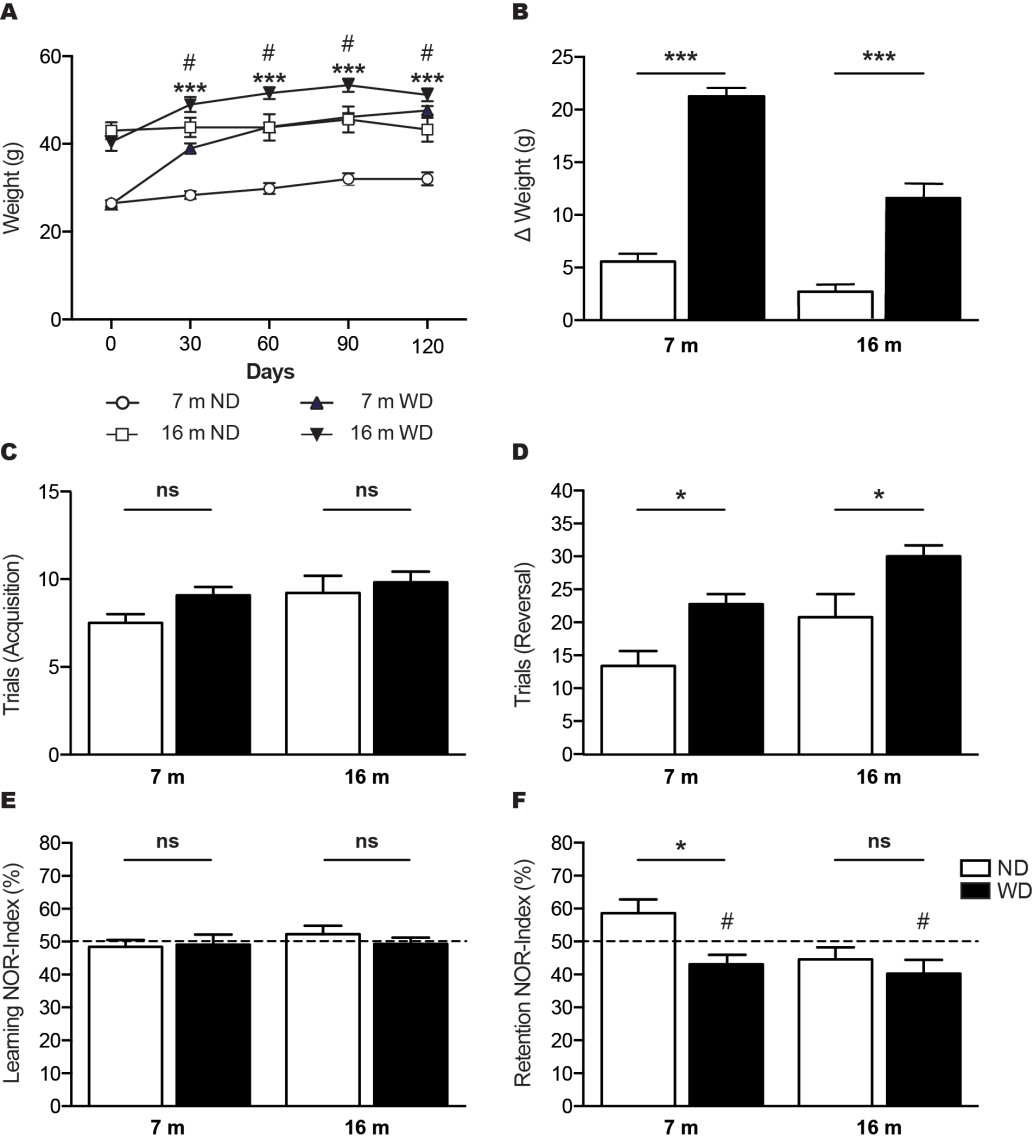
WD increases body weights and exacerbates age-induced cognitive decline in APPswe/PS1 mice Graph showing **A.** body weight follow up of 7 and 16 months old APPswe/PS1 mice fed with normal diet (ND) or “Western diet” (WD), which were weighted every 30 days during 4 months. Histograms showing **B.** body weight gains of animals following 4 months of ND or WD, in comparison to their initial weight. T-water maze behavioral test was used to assess left/right discrimination based on spatial learning and retention **C**., **D**. WD does not affect **C.** number of trials to reach criterion in the acquisition phase, but significantly exacerbates age-induced cognitive decline in APPswe/PS1 mice, as shown by **D.** increased number of trials to reach criterion in the reversal phase. The new object recognition (NOR) paradigm was used to assess memory and learning based on innate preference for novelty **E.**, **F.** WD does not affect **E.** NOR-indexes in the learning phase, but significantly exacerbates age-induced cognitive decline in APPswe/PS1 mice, as shown by **F.** reduced NOR-indexes in 7 months old mice in the retention phase. No significant changes were observed in 16 months old animals. NOR-indexes of 7 and 16 months old WD-fed mice are significantly below the **F.** theoretical value of 50%. Data are means ± SEM (*n* = 10-12 animals per group). Body weights; Unpaired T-test ***p < 0.0001, #*p* < 0.05, Two-way ANOVA *p* < 0.0001, Bonferroni post-hoc tests ***p < 0.001. T-maze acquisition phase; Two-way ANOVA *p* = 0.1066, Bonferroni post-hoc tests *p* > 0.05. T-maze reversal phase; Two-way ANOVA *p* = 0.0002, Bonferroni post-hoc tests *p < 0.05. NOR learning phase; Two-way ANOVA *p* = 0.6566, Bonferroni post-hoc tests *p* > 0.05. NOR retention phase; Two-way ANOVA *p* = 0.0117, Bonferroni post-hoc tests *p < 0.05, Paired *T*-test #*p* < 0.05.

The 3 (i.e. young) and 12 (i.e. aged) months old APPswe/PS1 mice fed during 4 months with a high fat “Western diet” (WD) or normal diet (ND), were weighed every 30 days. We observed a significant body weight gain following 30 days of WD, in 7 months old mice (i.e. young) (Unpaired *T*-test ***p < 0.0001) and 60 days in 16 months old mice (i.e. aged) (Unpaired *T*-test #*p* = 0.0206), which is maintained afterwards (Unpaired *T*-test ***p < 0.0001, #*p* < 0.05; Figure 1A). After 4 months of diet, 7 and 16 months old WD-fed animals showed significant body weight gains in comparison to their initial weight (Two-way ANOVA *p* < 0.0001, Bonferroni post-hoc tests ***p < 0.001; Figure 1B).

**Figure 2:**
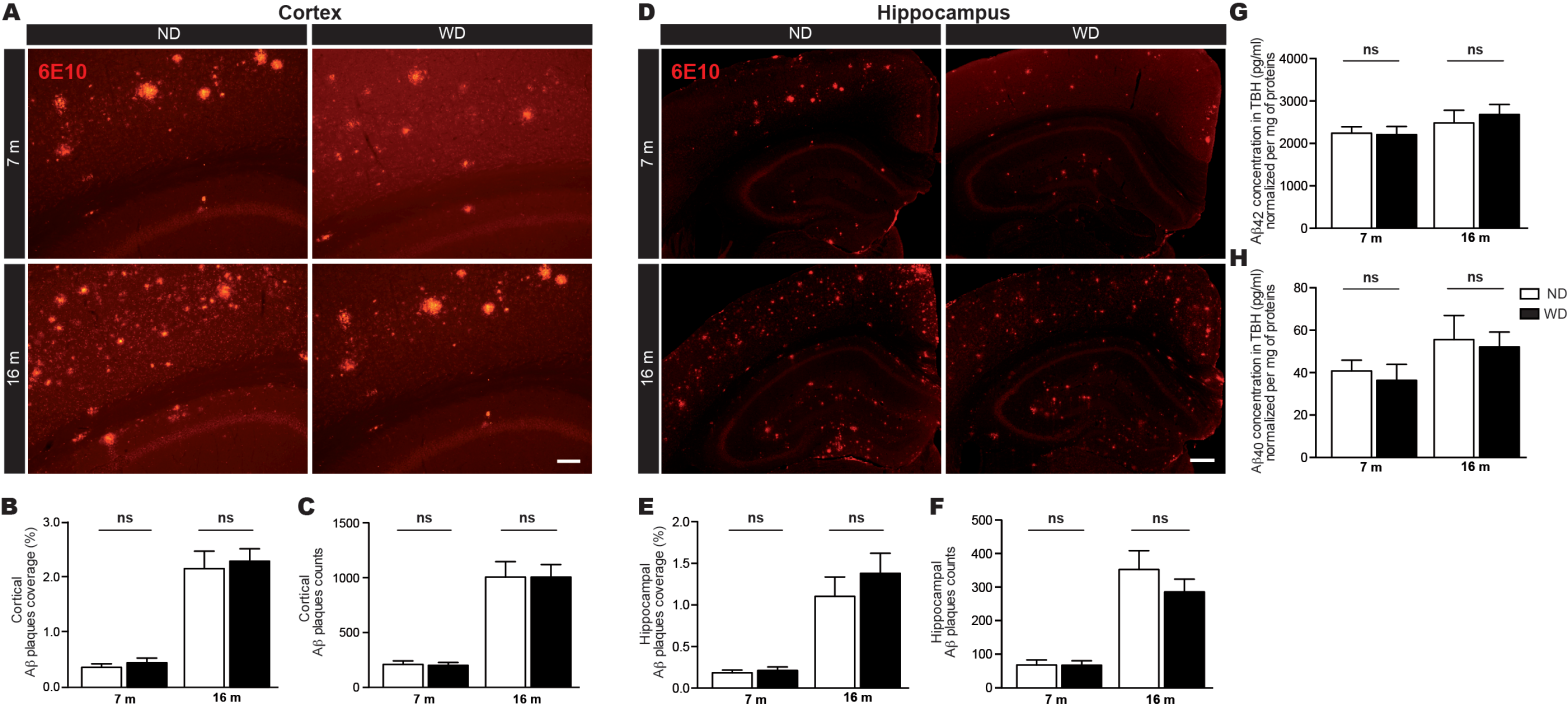
WD does not affect Aβ loads in brain parenchyma Fluorescent 6E10 immunostaining for Aβ plaques in the **A.** cortex or **D.** hippocampus of APPswe/PS1 mice. Quantification by stereological analyses of **B.**, **E.** size (μm2) and **C.**, **F.** number of 6E10-positive Aβ plaques in the **A.**-**C.** cortex and **D.**-**F.** hippocampus of 7 and 16 months old ND or WD-fed animals. No significant changes were observed neither in the cortex nor the hippocampus of WD-fed animals in comparison to ND-fed littermates, in terms of Aβ plaques **B.**, **E.** size and **C.**, **F.** number. Soluble Aβ species were measured by ELISA in brain (TBH) **G.**, **H.**. No significant changes were observed in soluble **G.** Aβ 1-42 or **H.** Aβ 1-40 levels normalized per mg of total proteins. Data are means ± SEM (*n* = 10-12 animals per group). Cortical Aβ plaques size; Two-way ANOVA *p* = 0.4309 and cortical Aβ plaques number; Two-way ANOVA *p* = 0.9430, Bonferroni post-hoc tests *p* > 0.05. Hippocampal Aβ plaques size; Two-way ANOVA *p* = 0.2531 and hippocampal Aβ plaques number; Two-way ANOVA *p* = 0.2115, Bonferroni post-hoc tests *p* > 0.05. Soluble Aβ 1-42; Two-way ANOVA *p* = 0.7177 and Aβ 1-40; Two-way ANOVA *p* = 0.6373, Bonferroni post-hoc tests *p* > 0.05. Scale bar A = 100μm. Scale bar D = 250μm.

Next, we evaluated the impact of WD on cognitive functions of APPswe/PS1 mice by performing two different neurobehavioral tests. First, we used a T-water maze behavioral paradigm, a left/right discrimination test that assesses specifically hippocampus-based spatial learning and retention (Figure 1C, 1D). The effect of WD during acquisition phase of T-water maze behavioral analysis was considered not significant, as any intergroup difference was observed (Two-way ANOVA *p* = 0.1066, Bonferroni post-hoc tests *p* > 0.05; Figure 1C). In fact, the four groups of mice took a similar number of trials before reaching criterion performance. However, during reversal phase, the analysis of trials to criterion revealed a highly significant effect of WD in both 7 and 16 months old mice (Two-way ANOVA *p* = 0.0002, Bonferroni post tests *p < 0.05; Figure 1D). Second, we used the new object recognition (NOR) paradigm that assesses the learning and memory based on innate preference for novelty (Figure 1E, 1F). No intergroup difference was observed during learning phase of NOR behavioral analysis and thus, the effect of WD was considered not significant (Two-way ANOVA *p* = 0.6566, Bonferroni post-hoc tests *p* > 0.05; Figure 1E). During retention phase, the analysis of recognition index (NOR-index), which represents the time spent exploring the novel object per total time, revealed a significant effect of WD in 7 months old mice (Two-way ANOVA *p* = 0.0117, Bonferroni post-hoc tests *p < 0.05; Figure 1F), while no significant changes were observed in 16 months old animals. In addition, NOR-index of 7 months old (Paired *T*-test #*p* = 0.0335; Figure 1F) and 16 months old (Paired T-test #*p* = 0.0417; Figure 2D) WD-fed animals were significantly below the theoretical value of 50%.

### WD does not affect soluble and insoluble Aβ loads in brain parenchyma

Afterwards, we evaluated the implication of parenchymal Aβ deposition in the cognitive deficits observed in WD-fed animals. For this purpose, the size and number of fluorescent 6E10-positive Aβ plaques were quantified by stereological analyses in the cortex (Figure 2A-2C) and hippocampus (Figure 2D-2F). Interestingly, our results revealed no significant changes neither in the cortex (Aβ plaque size; Two-way ANOVA *p* = 0.4309, Aβ plaque number; Two-way ANOVA *p* = 0.9430, Bonferroni post-hoc tests *p* > 0.05; Figure 2B, 2C respectively) nor the hippocampus (Aβ plaque size; Two-way ANOVA *p* = 0.2531, Aβ plaque number; Two-way ANOVA *p* = 0.2115, Bonferroni post-hoc tests *p* > 0.05; Figure 2E, 2F respectively) of 7 and 16 months old WD-fed animals in comparison to ND-fed littermates. Next, soluble Aβ 1-42 and Aβ1-40 species were measured by ELISA in brain (i.e. TBH). Similar to Aβ deposits, we did not observe any significant changes in brain soluble Aβ 1-42 (Two-way ANOVA *p* = 0.7177, Bonferroni post-hoc tests *p* > 0.05; Figure 2G) or Aβ 1-40 (Two-way ANOVA *p* = 0.6373, Bonferroni post-hoc tests *p* > 0.05; Figure 2H) levels in WD-fed animals.

### WD exacerbates age-associated decrease of MMP-9 enzymatic activity and reduces BDNF mRNA and protein levels in the brain, suggesting loss of synaptic plasticity

**Figure 3:**
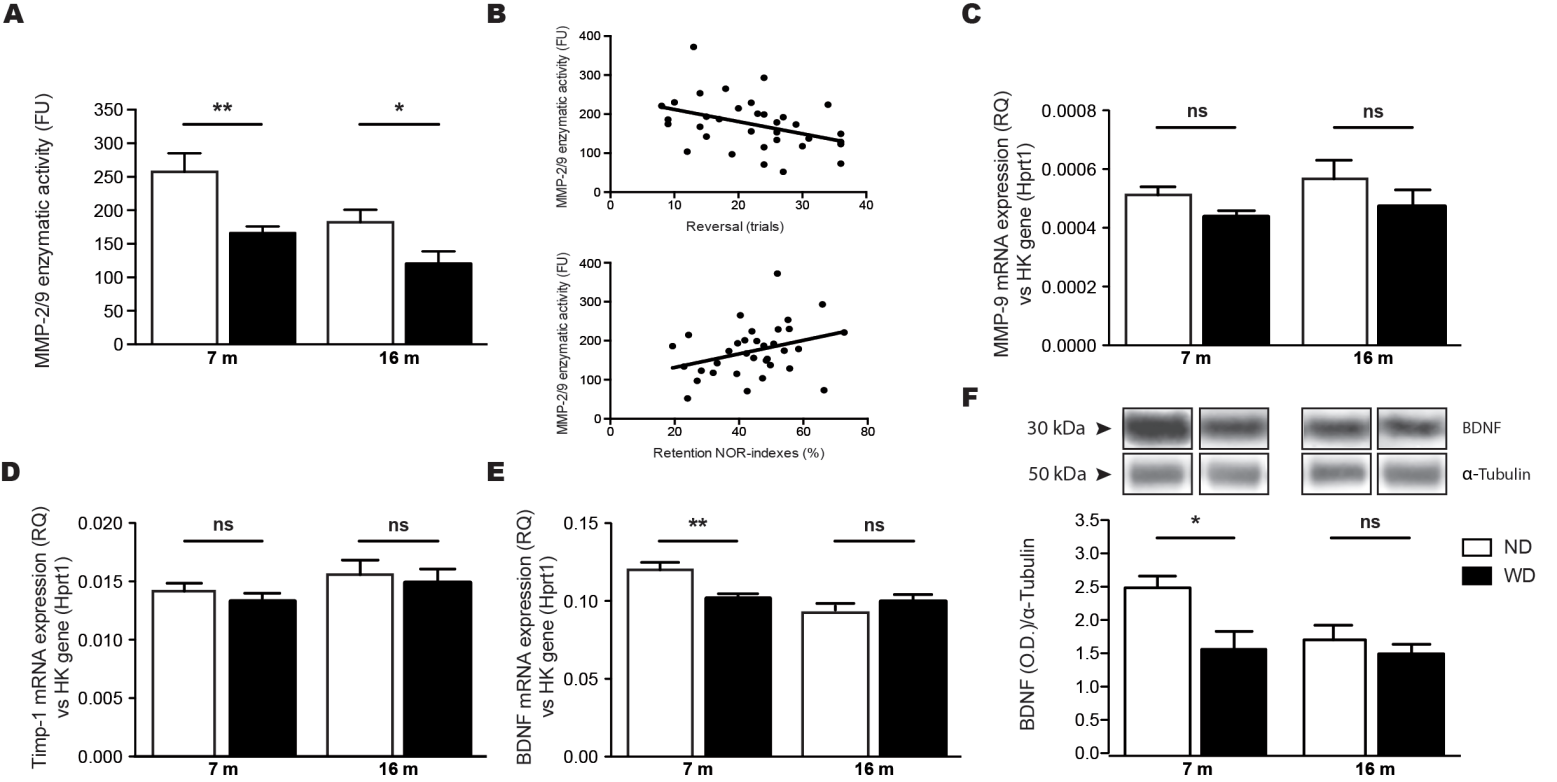
WD exacerbates age-associated decrease of MMP-9 enzymatic activity and reduces BDNF mRNA and protein levels in the brain Gelatin fluorescent zymography revealed a significant decrease of **A.** MMP-2/9 endogenous basal enzymatic activities normalized per mg of total proteins in brains (TBH) of 7 and 16 months old WD-fed APPswe/PS1 mice in comparison to ND-fed littermates. There are significant correlations between WD-induced exacerbation of age-associated reduced MMP 2/9 enzymatic activities and accelerated cognitive decline in 7 and 16 months old mice, as observed with the (**B**; upper panel) T-water maze paradigm and the (**B**; lower panel) NOR paradigm. Relative quantity (RQ) in brain samples of **C.** MMP-9, **D.** Timp-1 and **E.** BDNF mRNAs normalized per housekeeping gene expression (i.e. *hprt1*) revealing that WD does not induce significant changes in either MMP-9 or Timp-1 mRNAs levels, but significantly decreases *bdnf* gene expression in brains of 7 months old animals, in comparison to ND-fed littermates. BDNF protein levels were measured by Western blot analysis in TBH, which are represented by cropped blots. WD significantly reduced **F.** BDNF protein levels normalized with tubulin in brains of 7 months old WD-fed animals, in comparison to ND-fed littermates. Data are means ± SEM (*n* = 10-12 animals per group). MMP-2/9 enzymatic activities; Two-way ANOVA *p* = 0.0002, Bonferroni post-hoc tests **p < 0.01, *p < 0.05. T-maze correlation; Spearman r = −0.4254, *p = 0.0121. NOR correlation; Spearman r = 0.3510, *p = 0.0418. MMP-9 mRNA; Two-way ANOVA *p* = 0. 0775, Bonferroni post-hoc tests *p* > 0.05. Timp-1 mRNA; Two-way ANOVA *p* = 0.4179, Bonferroni post-hoc tests *p* > 0.05. BDNF mRNA; Two-way ANOVA *p* = 0.0019, Bonferroni post-hoc tests **p < 0.01. BDNF protein; Two-way ANOVA *p* = 0.0354, Bonferroni post-hoc tests *p < 0.05.

As mentioned, MMP-9 contributes to the process of synaptic plasticity. Therefore, MMP-2/9 basal enzymatic activities were measured in brain by using gelatin fluorescent zymography. Interestingly, WD significantly reduced the endogenous activity of MMP-2/9 in brains of 7 and 16 months old WD-fed animals, in comparison to their ND-fed littermates (Two-way ANOVA *p* = 0.0002, Bonferroni post-hoc tests **p < 0.01, *p < 0.05; Figure 3A). In addition, this reduction in MMP-2/9 correlated with the observed cognitive decline in all groups of mice (Figure 3B), reported in the T-water maze paradigm (Spearman r = −0.4254, *p = 0.0121; upper panel) and the NOR paradigm (Spearman r = 0.3510, *p = 0.0418; lower panel). Afterwards, we performed qPCR quantifications of MMP-9, TIMP metallopeptidase inhibitor 1 (Timp-1, a tissue inhibitor of MMP-9) and BDNF mRNA levels in brain samples, which are expressed as relative quantity (RQ) normalized per housekeeping gene expression (i.e. *hprt1*). Interestingly, we observed no significant changes in either MMP-9 (Two-way ANOVA *p* = 0. 0775, Bonferroni post-hoc tests *p* > 0.05; Figure 3C) or Timp-1 (Two-way ANOVA *p* = 0.4179, Bonferroni post-hoc tests *p* > 0.05; Figure 3D) mRNA levels in brains of 7 and 16 months old animal groups. However, we observed a significant decrease of *bdnf* gene expression in brains of 7 months old WD-fed animals, comparatively to ND-fed littermates (Two-way ANOVA *p* = 0.0019, Bonferroni post-hoc tests **p < 0.01; Figure 3E), which was accompanied by reduced BDNF protein levels in brains of 7 months old WD-fed animals, comparatively to ND-fed littermates (Two-way ANOVA *p* = 0.0354, Bonferroni post-hoc tests *p < 0.05; Figure 3F).

### WD increases frequencies of blood-circulating monocytes and hematopoietic stem cells in the bone marrow, which is accompanied by increased MCP-1 production

**Figure 4:**
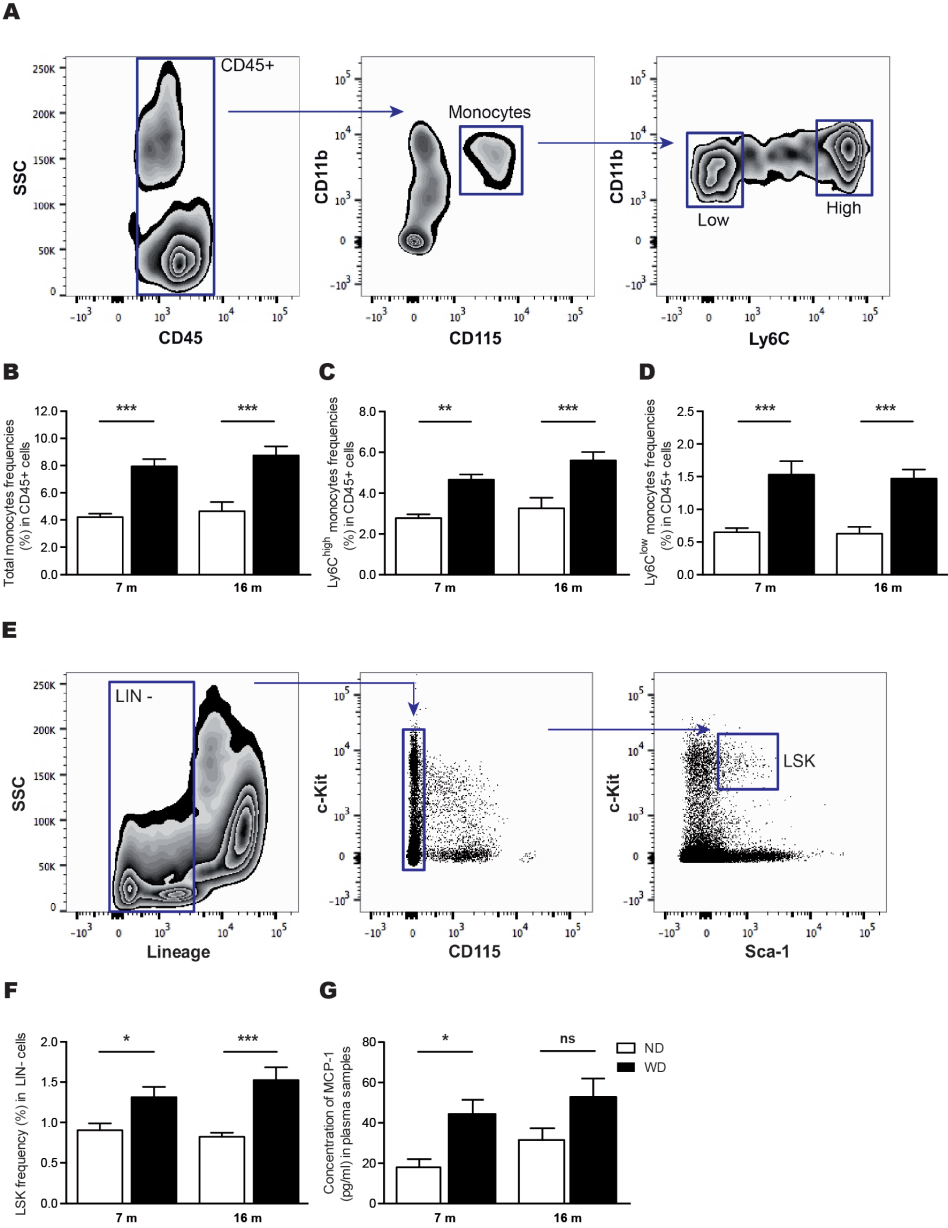
WD increases frequencies of blood-circulating monocytes and hematopoietic stem cells in the bone marrow, which is accompanied by increased MCP-1 levels in the plasma Gating strategy **A.** used to investigate blood-circulating monocyte (CD11b^+^CD115^+^) populations in leukocytes (CD45^+^), namely the pro-inflammatory subset (Ly6C^High^) and patrolling subset (Ly6C^Low^). Following 4 months of diet, flow cytometry analyses revealed that **B.** total monocytes, as well as **C.** Ly6C^High^ and **D.** Ly6C^Low^ monocytes relative frequencies expressed in CD45^+^ cells are significantly increased in WD-fed animals in comparison to ND-fed littermates. Gating strategy **E.** used to investigate hematopoietic stem cells levels (HSC or LSK; Lin^−^Sca-1^+^c-Kit^+^) in the bone marrow. Flow cytometry analyses revealed that **F.** HSC relative frequencies expressed in lineage-negative (Lin^−^) cells are significantly increased in WD-fed animals in comparison to ND-fed littermates. Quantification of **G.** MCP-1 in plasma samples revealed an increase in MCP-1 levels in 7 months-old WD-fed animals in comparison to ND-fed littermates, while no significant changes were observed in 16 months-old animals. Data are means ± SEM (*n* = 10-12 animals per group). Monocyte populations; Two-way ANOVA *p* < 0.0001, Bonferroni post-hoc tests **p < 0.01, ***p < 0.001. HSC; Two-way ANOVA *p* < 0.0001, Bonferroni post-hoc tests *p < 0.05, ***p < 0.001. MCP-1; Two-way ANOVA *p* < 0.0047, Bonferroni post-hoc tests *p < 0.05.

Previous reports have been demonstrated that WD contributes to systemic inflammation [21, 32]. After 4 months of diet, we observed by flow cytometry a significant increase of monocyte populations, including both Ly6C^High^ and Ly6C^Low^ subsets [9] (Figure 4A), in blood circulation of 7 and 16 months old WD-fed animals (Two-way ANOVA *p* < 0.0001, Bonferroni post-hoc tests **p < 0.01, ***p < 0.001; Figure 4B-4D), in comparison to ND-fed littermates. Moreover, since WD-induced hyperlipidemia modulates the proliferation of hematopoietic stem cells (HSC) [21], which are defined as Lin^−^Sca-1^+^c-Kit^+^ cells (LSK) [14] (Figure 4E), we performed flow cytometry analyses in freshly isolated bone marrow cells following 4 months of diet. As expected, we observed a significant increase of HSC levels in bone marrow of 7 and 16 months old WD-fed animals in comparison to ND-fed littermates (Two-way ANOVA *p* < 0.0001, Bonferroni post-hoc tests *p < 0.05, ***p < 0.001; Figure 4F). In addition, we observed significant increased levels of MCP-1 in the plasma of 7 months old WD-fed animals (Two-way ANOVA *p* < 0.0047, Bonferroni post-hoc tests *p < 0.05; Figure 4G) in comparison to ND-fed animals, coinciding with WD-induced monocytosis.

### LDL oxidation is increased in the plasma of APPswe/PS1 mice fed with WD

**Figure 5:**
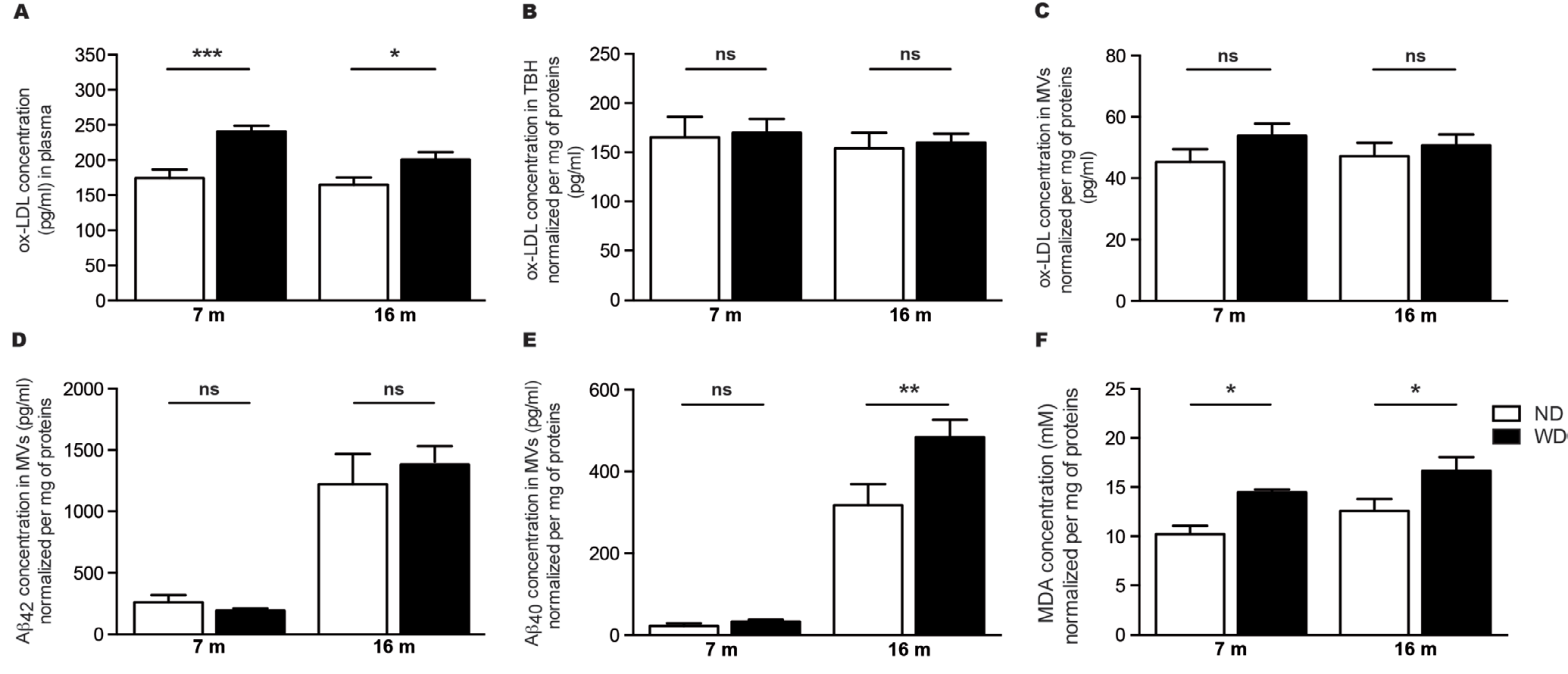
WD increases ox-LDL levels in the blood circulation and exacerbates soluble Aβ 1-40 and MDA accumulation in cerebral microvasculature WD-induced oxidative stress was assessed *via* the quantification of oxidized-LDL (ox-LDL) levels in **A.** plasma and **B**., **C**. brain samples by ELISA. Blood ox-LDL levels **A.** are significantly increased in WD-fed APPswe/PS1 mice in comparison to ND-fed littermates. No changes were observed in brain ox-LDL levels normalized per mg of total proteins, either in **B.** TBH or **C.** isolated cerebral MVs of WD-fed animals in comparison to ND-fed littermates. Quantification of soluble **D.** Aβ 1-42 and **E.** Aβ 1-40 levels normalized per mg of total proteins in isolated cerebral MVs by ELISA revealed a significant increase of soluble Aβ 1-40 levels in 16 months old WD-fed animals in comparison to ND-fed littermates. No changes were observed in Aβ 1-42 levels. Quantifications of **F.** malondialdehyde (MDA) normalized per mg of total proteins in isolated cerebral MVs by a TBARS assay revealed a significant increased of MDA levels (i.e. oxidative stress) in 7 and 16 months old WD-fed animals, in comparison to ND-fed littermates. Data are means ± SEM (*n* = 10-12 animals per group). Blood ox-LDL; Two-way ANOVA *p* < 0.0001, Bonferroni post-hoc tests ***p < 0.001, *p < 0.05. TBH ox-LDL; Two-way ANOVA *p* = 0.7284 and MVs ox-LDL; Two-way ANOVA *p* = 0.1402, Bonferroni post-hoc tests *p* > 0.05. Aβ 1-40; Two-way ANOVA *p* = 0.0178, Bonferroni post-hoc tests **p < 0.01. Aβ 1-42; Two-way ANOVA *p* = 0.7810, Bonferroni post-hoc tests *p* > 0.05. MDA; Two-way ANOVA *p* = 0.0014, Bonferroni post-hoc tests *p < 0.05.

As WD-induced hyperlipidemia increases blood LDL levels, which are highly susceptible to oxidation in inflammatory conditions [21, 32], we first investigated whether ox-LDL levels are increased in plasma samples of WD-fed APPswe/PS1 mice. Interestingly, we observed a significant augmentation of ox-LDL levels in plasmas of 7 and 16 months old WD-fed animals, in comparison to ND-fed littermates (Two-way ANOVA *p* < 0.0001, Bonferroni post-hoc tests ***p < 0.001, *p < 0.05; Figure 5A). However, we did not observe any changes in ox-LDL levels neither in TBH (Two-way ANOVA *p* = 0.7284, Bonferroni post-hoc tests *p* > 0.05; Figure 5B) nor isolated cerebral microvessels (MVs) (Two-way ANOVA *p* = 0.1402, Bonferroni post-hoc tests *p* > 0.05; Figure 5C) of WD-fed animals in comparison to ND-fed littermates.

### WD increases vascular levels of soluble Aβ 1-40 and malondialdehyde, without affecting the integrity of the BBB

Since no changes were previously observed in soluble Aβ species in brain parenchyma, we investigated whether WD could specifically increase soluble Aβ species levels in cerebral microvasculature. For this purpose, soluble Aβ 1-42 and Aβ 1-40 were measured by ELISA in isolated cerebral MVs. Interestingly, we observed a significant increase of soluble Aβ 1-40 levels (Two-way ANOVA *p* = 0.0178, Bonferroni post-hoc tests **p < 0.01; Figure 5E), but not Aβ 1-42 (Two-way ANOVA *p* = 0.7870, Bonferroni post-hoc tests *p* > 0.05; Figure 5D), in cerebral microvasculature of 16 months old WD-fed mice in comparison to ND-fed littermates, while only subtle changes were observed in 7 months old animals.

Next, we investigated whether WD-induced ox-LDL may affect oxidative stress specifically in cerebral microvasculature. For this purpose, we chose MDA as a marker of occurring oxidative stress [33]. MDA levels were measured in cerebral MVs by using the TBARS assay. Interestingly, we observed a significant increase of MDA levels in cerebral microvasculature of both 7 and 16 months old WD-fed animals (Two-way ANOVA *p* = 0.0014, Bonferroni post-hoc tests *p < 0.05; Figure 5F), in comparison to ND-fed littermates.

**Figure 6:**
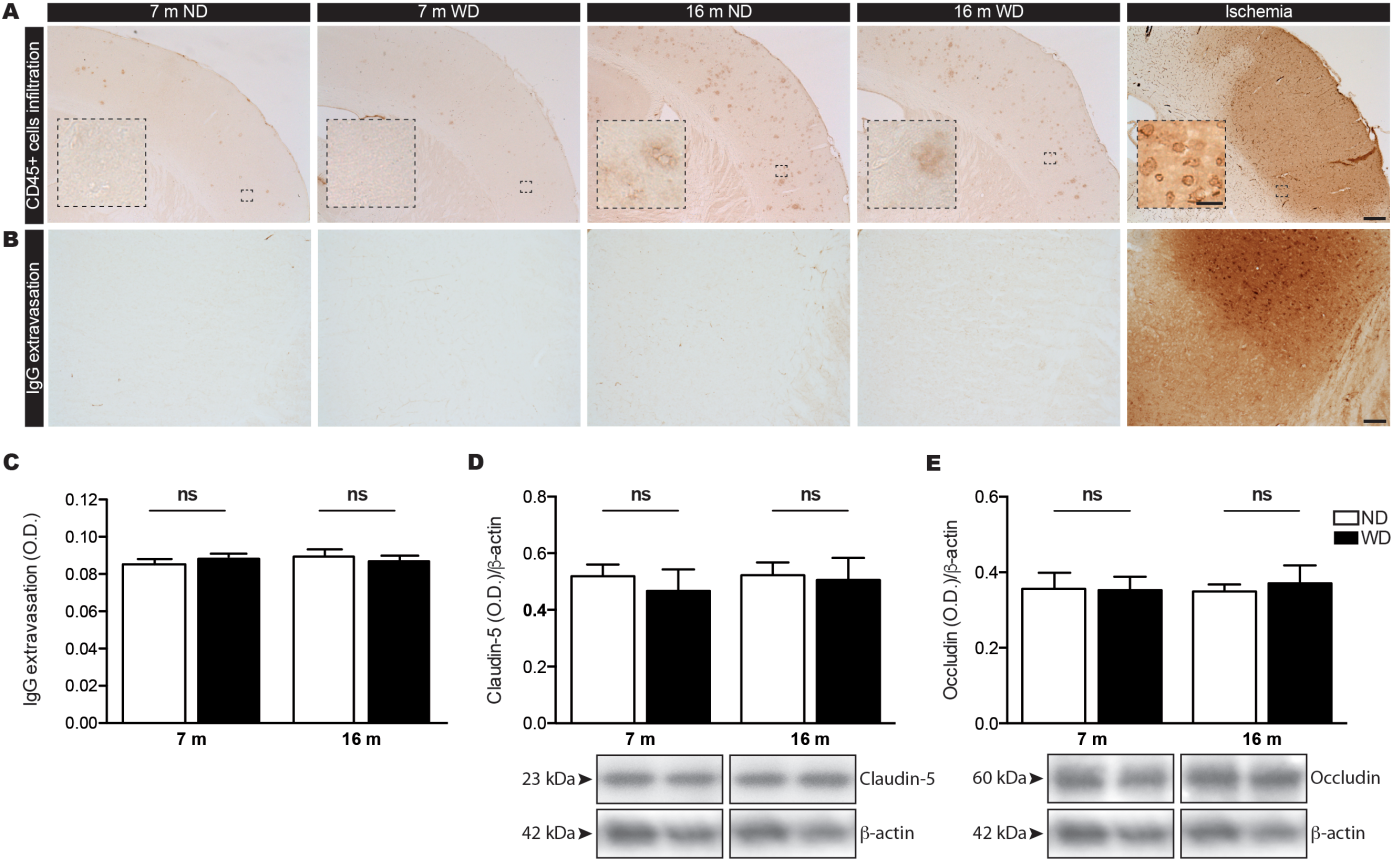
WD does not induce infiltration of immune cells or IgG extravasation in the brain parenchyma since the BBB integrity is not compromised CD45 histochemical immunostaining was performed to assess infiltration of immune cells in brain parenchyma, which are designated as CD45^+^ cells. WD does not induce brain infiltration as no **A.** CD45^+^ cells were observed in the brain parenchyma of both 7 and 16 months old ND or WD-fed animals, comparatively to positive control animal (ischemia). Immunoglobulins (IgG) immunostaining was performed to assess BBB integrity. No changes were observed in **B.** IgG extravasation as measured by **C.** densitometric analysis in the brain parenchyma of both 7 and 16 months old ND or WD-fed animals, in comparison to positive control animal (ischemia). Pictures showing representative cortices for each experimental group and positive control (ischemia). The expression levels of tight junction proteins namely **D.** claudin-5 and **E.** occludin were measured by Western blot analyses in TBH, which are represented by cropped blots. No significant changes were observed in either claudin-5 or occludin protein levels normalized with actin in TBH of WD-fed animals, in comparison to ND-fed littermates. Data are means ± SEM (*n* = 10-12 animals per group). IgG extravasation; Two-way ANOVA *p* = 0.9593, Bonferroni post-hoc tests *p* > 0.05. Claudin-5; Two-way ANOVA *p* = 0.5922, Bonferroni post-hoc tests *p* > 0.05. Occludin; Two-way ANOVA *p* = 0.8168, Bonferroni post-hoc tests *p* > 0.05). Scale bar A = 250μm. Scale bar A (Zoom in) = 25μm. Scale bar B = 100μm.

Finally, we investigated whether these events affect BBB physical integrity, by evaluating infiltration of immune cells and extravasation of immunoglobulins (IgG) in brain parenchyma. As such, we analyzed the infiltration of circulating immune cells into the brain by examining CD45 expression (i.e. leukocyte marker), where CD45^+^ cells in brain parenchyma are designated as infiltrated cells [34, 35]. As expected, we did not observe any infiltration of immune cell in brains of 7 and 16 months old animal groups (Figure 6A), comparatively to the positive control (i.e. ischemic brain). Additionally, we did not detect any IgG extravasation in both 7 and 16 months old animal groups (Figure 6B), in comparison to positive control (ischemic brain), as measured by densitometric analysis (Two-way ANOVA *p* = 0.9593, Bonferroni post-hoc tests *p* > 0.05; Figure 6C). Afterwards, we assessed the expression of tight junction proteins in brain, namely claudin-5 (Figure 6D) and occludin (Figure 6E), by Western blot analyses. We did not observed any changes in the protein expression level of claudin-5 (Two-way ANOVA *p* = 0.5922, Bonferroni post-hoc tests *p* > 0.05; Figure 6D) and occludin (Two-way ANOVA *p* = 0.8168, Bonferroni post-hoc tests *p* > 0.05; Figure 6E) in 7 and 16 months old WD-fed animals, in comparison to ND-fed littermates.

### WD induces pericytes dysfunction, thus altering BBB functional properties

**Figure 7:**
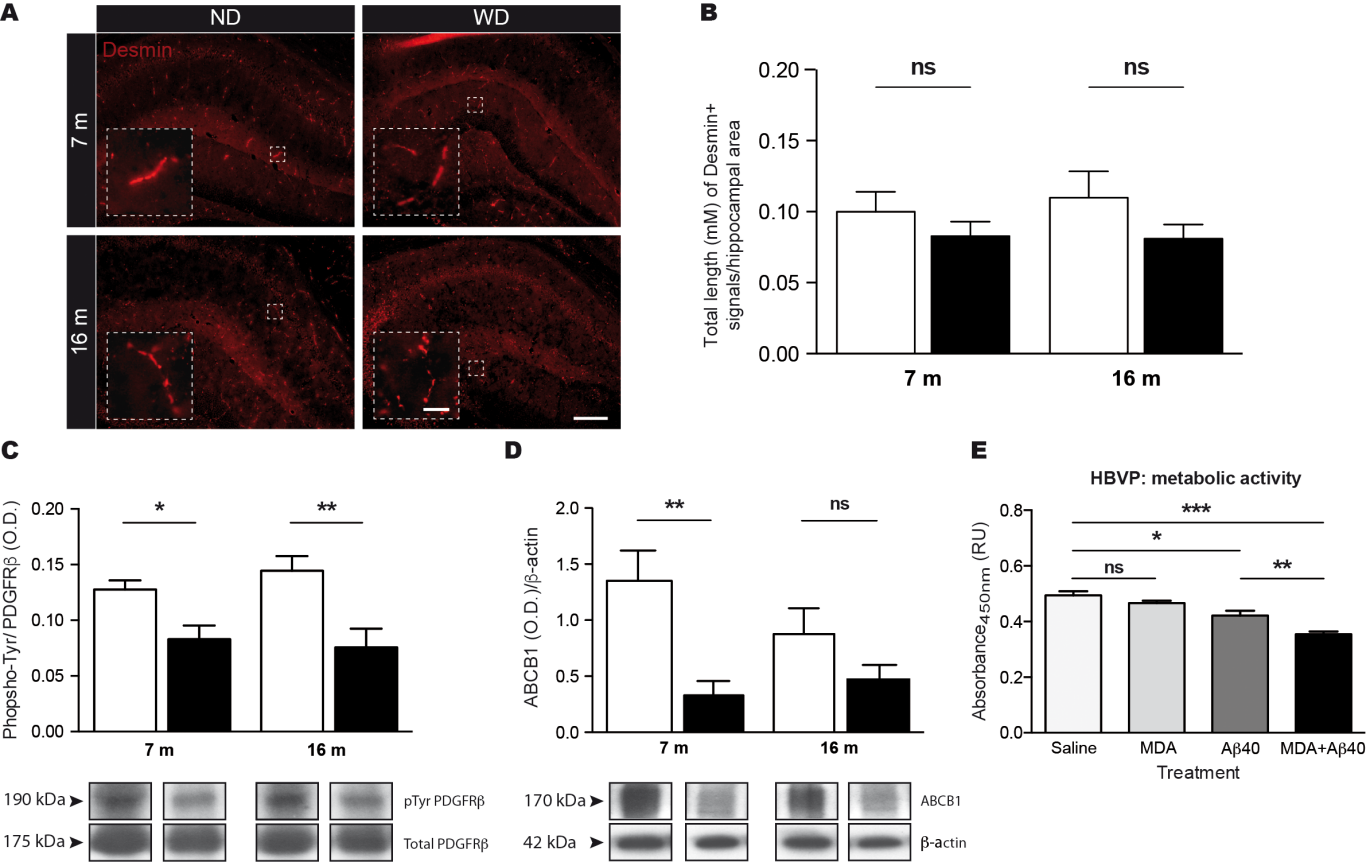
WD induces pericyte dysfunction without affecting their viability, which is associated to altered BBB functionality The viability of pericytes was assessed *via* **A.** fluorescent immunostaining for Desmin. Pictures showing representative hippocampus for each experimental group. Quantification of pericyte coverage by stereological analysis expressed as **B.** total length (μm) of hippocampal Desmin-positive cerebral MVs. No significant changes in Desmin-positive MVs were observed in WD-fed animals in comparison to ND-fed littermates. Immunoprecipitation of total platelet-derived growth factor receptor-β (PDGFRβ) proteins was performed in brain (TBH) prior to Western blot analysis for phospho-tyrosine, which is represented by cropped blots. Quantifications of **C.** phospho-tyrosine in pooled down PDGFRβ protein samples normalized by total PDGFRβ proteins revealed a significant reduction in phospho-tyrosine levels in brain of WD-fed animals, in comparison to ND-fed littermates. Quantifications of **D.** ABCB1 protein levels in cerebral MVs revealed a significant reduction in brain of 7 months old WD-fed animals, in comparison to ND-fed littermates. Samples derive from the same experiment and blots were performed in parallel. Human brain vascular pericytes (HBVP) were incubated with 0.5 μg of soluble Aβ 1-40 and/or 1 mM of MDA for 24 h prior to metabolic activity assay. Absorbance quantifications revealed significant reductions in **E.** metabolic activities of HBVP exposed to Aβ 1-40 monomers with and without MDA, while no significant changes were observed in MDA-treated cells alone, in comparison to vehicle-treated cells. MDA significantly worsens soluble Aβ 1-40-induced decrease in metabolic activities of HBVP. Data are means ± SEM (*n* = 10-12 animals per group (*in vivo*), *n* = 12 wells per group (*in vitro*)). Desmin; Two-way ANOVA *p* = 0.0843, Bonferroni post-hoc tests *p* > 0.05. Phosphorylated PDGFRβ; Two-way ANOVA *p* = 0.0003, Bonferroni post-hoc tests *p < 0.05, **p < 0.01. ABCB1; Two-way ANOVA *p* = 0.0023, Bonferroni post-hoc tests **p < 0.01. Aβ 1-40+MDA *vs*. Vehicle; Unpaired *T*-test ***p < 0.0001. Aβ 1-40 *vs*. Vehicle; Unpaired *T*-test *p = 0.0173. MDA *vs*. Vehicle; Unpaired *T*-test *p* > 0.05. Aβ 1-40+MDA *vs*. Aβ 1-40; Unpaired *T*-test **p = 0.0058. Scale bar A = 25μm. Scale bar A (Zoom in) = 100μm.

Due to their central role in cerebral microvasculature function, and more precisely BBB function, we next investigated the impact of soluble Aβ 1-40 and MDA accumulation in cerebral MVs on pericyte function. For this purpose, fluorescent immunostaining for Desmin was used to assess pericytes of cerebral microvasculature [36] (Figure 7A). Interestingly, quantifications of Desmin-positive cerebral MVs by stereological analysis in hippocampus did not depict any differences in 7 and 16 months old WD-fed animals (Two-way ANOVA *p* = 0.0843, Bonferroni post-hoc tests *p* > 0.05; Figure 7B) in comparison to ND-fed littermates, suggesting that WD does not affect viability of pericytes.

Next, we investigated whether WD could compromise the function of pericytes, by assessing the phosphorylation (i.e. activation) of platelet-derived growth factor receptor β (PDGFRβ), a key receptor involved in promoting pericyte function [37]. For this purpose, we used an immunoprecipitation technique to pool PDGFRβ in the brain and evaluate its tyrosine phosphorylation by Western blot analysis. Interestingly, we observed a significant reduction of phosphorylated PDGFRβ levels in the brain of 7 and 16 months old WD-fed animals (Two-way ANOVA *p* = 0.0003, Bonferroni post-hoc tests *p < 0.05, **p < 0.01; Figure 7C), in comparison to ND-fed littermates. In addition, we investigated whether pericyte dysfunction could affect BBB functionality. Since ABCB1 is a marker of BBB transport function [26], we assessed by Western blot analysis its protein expression levels in cerebral MVs. Interestingly, we observed reduced ABCB1 protein levels in brains of 7 months old WD-fed animals, in comparison to ND-fed ones (Two-way ANOVA *p* = 0.0023, Bonferroni post-hoc tests **p < 0.01;Figure 7D), indicating that although BBB physical properties are not altered, WD induces alterations in BBB functional properties.

Finally, in order to address the direct contribution of soluble Aβ 1-40 and MDA accumulation in cerebral microvasculature dysfunction, we investigated the direct effects of these molecules on human brain vascular pericytes (HBVP) *in vitro*. For this purpose, 1×10^4^ HBVP were incubated with 0.5 μg of Aβ 1-40 monomers with and without 1 mM of MDA for 24 h prior to metabolic activity assay (XTT cell viability). Importantly, we observed a significant reduction of metabolic activity of HBVP exposed to both soluble Aβ 1-40 and MDA (Unpaired *T*-test ***p < 0.0001; Figure 7E), in comparison to vehicle-treated cells. This reduction, required the presence of Aβ 1-40, as soluble Aβ 1-40-treated alone exhibited a significant decrease in their metabolic activity in comparison to vehicle-treated cells (Unpaired *T*-test *p = 0.0173; Figure 7E), while MDA-treated cells alone did not exhibit any change in their metabolic activity compared to vehicle-treated cells (Unpaired *T*-test *p* = 0.1956; Figure 7E). These observations indicate that soluble Aβ 1-40 decreases the metabolic activity of HBVP, which is worsened by MDA (Unpaired *T*-test **p = 0.0058; Figure 7E), suggesting direct contribution of soluble Aβ 1-40 and MDA in pericyte dysfunction, supporting our previous *in vivo* results.

## DISCUSSION

This study aims to clarify the synergistic role of age and high fat diet (i.e. WD) in the progression of AD-related pathology in APPswe/PS1 mice. Neurobehavioral analyses revealed an acceleration of age-induced cognitive decline in WD-fed animals, which was not associated to Aβ plaque deposition and soluble Aβ abundance in brain parenchyma. However, the cognitive decline was accompanied with an exacerbated decrease of MMP-9 enzymatic activity and reduction of BDNF mRNA and protein levels in brain, which suggest loss of synaptic plasticity. In the periphery, we observed higher levels of blood-circulating monocytes, MCP-1 and ox-LDL in the plasma of WD-fed mice, indicating a severe systemic inflammation in these animals. Furthermore, we observed an age-induced augmentation of soluble Aβ 1-40 levels and increased oxidative stress in cerebral microvasculature, which were exacerbated by WD. Importantly, the increased levels of soluble Aβ 1-40 and the exacerbation of oxidative stress in cerebral microvasculature of WD-fed animals jointly contributed to pericyte dysfunction, which was confirmed *in vitro*. It is noteworthy to mention that BBB physical properties were similar among groups indicating that, in our study, age and WD affected mainly cerebral microvasculature function without affecting BBB integrity. Taken together, our results unravel new insights into the role of age and WD in AD pathogenesis and progression.

**Figure 8:**
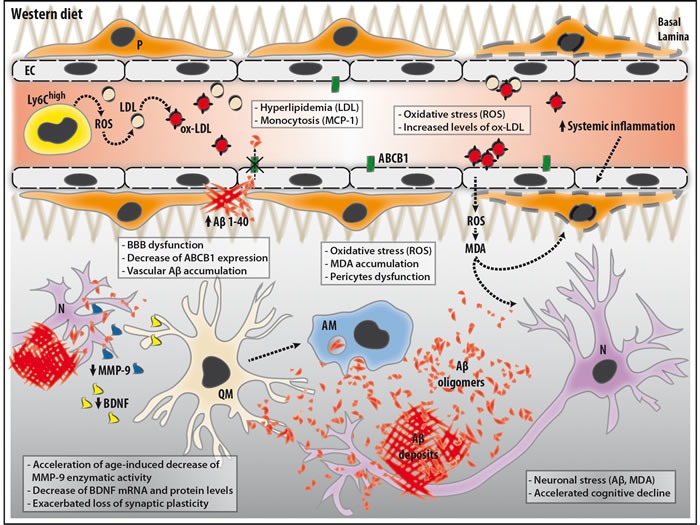
Schematic illustration of the potential mechanisms involved in our observations in APPswe/PS1 mice following WD WD increases the blood levels of low-density lipoproteins (LDL), which are highly susceptible to oxidation. WD increases the frequency of blood-circulating monocytes, namely pro-inflammatory monocytes (Ly6CHigh), which facilitate the production of pro-inflammatory molecules, such as reactive oxygen species (ROS), thus contributing to systemic inflammation. WD-induced ROS generation promotes the formation of oxidized-LDL (ox-LDL) in the blood circulation. Blood ox-LDL interacts with cerebral microvasculature and induces the production and release of ROS, namely peroxynitrite (ONOO-), by endothelial cells (EC) in the perivascular space and brain parenchyma. This leads to the formation of malondialdehyde (MDA), which is one of the most prevalent lipid peroxidation products during oxidative stress. In the brain, MDA accumulation can directly impair neurons (N) and pericytes (P), and contribute to neuroinflammation. In parallel, MDA contribute to WD-induced impairment of pericytes, thereby leading to blood-brain barrier (BBB) dysfunction, which is translated by a decreased expression of ATP-binding cassette sub-family B member 1 (ABCB1) on the cerebral microvasculature, without altering its physical structure and permeability. ABCB1 contributes to the clearance of Aβ, and its reduced expression promotes the accumulation of Aβ aggregates within cerebral microvasculature. As a vicious circle, Aβ aggregates contribute to neuronal stress and neuroinflammation *via* the activation of quiescent microglia (QM). Activated microglia (AM) are involved in various immune responses such as Aβ phagocytosis. However, AM prolonged exposure to Aβ aggregates and the impaired microenvironment caused by BBB dysfunction lead to microglial dysfunction. This reduces the secretion of brain-derived neurotrophic factor (BDNF) and decreases enzymatic activity of matrix metalloproteinase-9 (MMP-9), which are both involved in synaptic plasticity. Altogether, these changes induced either directly or indirectly by WD can contribute to the cognitive deficits observed in APPswe/PS1 mice.

Although the impact of high fat diet on AD-related pathology has been studied in various animal models and conditions [19], the mechanisms that link risk factors to AD pathogenesis remains unclear. In our study, 3 (i.e. young) and 12 (i.e. aged) months old APPswe/PS1 mice were fed with a widespread adjusted calories high fat diet, which presents the features of a “Western diet” (WD) [38]. After 4 months of diet, T-maze and NOR neurobehavioral tests revealed that cognitive deficits of young WD-fed mice were similar to aged ND-fed littermates, suggesting that WD significantly accelerated cognitive decline in young APPswe/PS1 mice. In addition, WD exacerbated cognitive deficits of aged WD-fed mice in comparison to ND-fed littermates, as reported by T-maze neurobehavioral test. Interestingly, these results were not associated to Aβ plaque deposition and soluble Aβ abundance in brain parenchyma, since no changes were detected in WD-fed animals in comparison to ND-fed littermates. These observations are not unexpected since several studies reported that Aβ plaques do not necessarily correlate with cognitive deficits, proposing a “toxic Aβ oligomer” hypothesis [2]. However, no changes were observed in soluble Aβ 1-42 and 1-40 levels in brain parenchyma of WD-fed mice in comparison to ND-fed animals, which does not exclude the contribution of oligomeric Aβ in cognitive decline (Figure 8). While our results are in line with a number of previously published reports [20, 39], others demonstrated an increase in insoluble and soluble Aβ loads in the brain parenchyma following an high fat diet, namely in 3xTg-AD mice [40]. These discrepancies highlight the importance of the experimental settings that include, diet composition (e.g. various dietary factors and concentrations), exposure time and AD transgenic animal model used and its susceptibility to the different diets [19]. Taken together, our results indicate that other factors, which are not necessarily dependent of parenchymal Aβ, are involved.

As mentioned, cognitive functions tightly depend on healthy neuronal circuitries and synaptic plasticity, a physiological process that is essential for dendrite remodeling and neuronal connections, which implicate the enzymatic activity of MMP-9 [4, 5] and BDNF secretion [6, 41]. Consumption of WD in rats affects synaptic plasticity, thus leading to altered neuronal morphology, dendritic integrity and blood vessel structure in hippocampus [42]. Here we showed an age-dependent decrease of cerebral MMP-2/9 endogenous enzymatic activities and BDNF mRNA and protein levels that suggest loss of synaptic plasticity, which is exacerbated by WD. Interestingly, this reduction of MMP-2/9 enzymatic activity is not associated to downregulation of *mmp9* or upregulation of *timp1* gene expression, but is accompanied by decreased *bdnf* gene expression, a long-term potentiation (LTP)- related gene [6]. Importantly, it has been reported that BDNF can modulate MMP-9 mRNA and protein levels, as well as its enzymatic activity [41], suggesting that WD-induced decrease of MMP-9 enzymatic activity may be associated to reduced *bdnf* gene expression and protein levels in brain. These results suggest that WD exacerbates the age-associated loss of synaptic plasticity, suggesting its contribution in the acceleration of the cognitive decline in APPswe/PS1 mice (Figure 8).

In order to clarify the mechanism involved in this WD-induced acceleration of cognitive deficits, we evaluated systemic inflammation, which has been linked to neuroinflammation, a phenomenon associated to AD pathogenesis [43]. Arising from the hematopoietic system composed by self-renewal HSC located in the bone marrow, monocytes are involved in secretion of soluble mediators such as pro-inflammatory molecules (e.g. MCP-1) [9, 44]. As such, WD has been demonstrated to promote systemic inflammation by activated immune cells in the periphery, namely monocytes [22]. In our study, we observed that WD significantly increases monocyte populations, including both Ly6C^High^ and Ly6C^Low^ subsets, in the blood of both young and aged APPswe/PS1 mice. A previous study has reported that Ly6C^High^ monocyte levels are increased in hypercholesterolemic ApoE-deficient mice consuming a high fat diet, suggesting that lipids influence the expansion of circulating myeloid cells [13]. As such, we evaluated whether WD could modulate the proliferation of HSC in APPswe/PS1 mice, which have been reported in other animal models [21]. As expected, we observed a significant increase of HSC frequencies in the bone marrow of young and aged WD-fed animals in comparison to ND-fed littermates, thus suggesting that WD increased blood-circulating monocyte levels, mainly by over-stimulating HSC proliferation in the bone marrow. In addition, we observed significant increased levels of MCP-1 in the plasma of young WD-fed animals, coinciding with monocytosis. This suggests that increased blood-circulating monocytes levels may lead to higher secretion of MCP-1, thus promoting monocyte mobilization into the blood circulation [45]. Interestingly, in our context, such a systemic inflammation does not trigger infiltration of immune cells in brain parenchyma since BBB permeability is not compromised. Our observations corroborate the role of WD in the development of chronic systemic inflammation, in APPswePS1 mice (Figure 8).

As such, chronic systemic inflammation exerts its detrimental effects *via* oxidative stress and ROS production [46, 47], which is also linked to AD pathogenesis [48, 49]. This phenomenon leads to the oxidation of various bioactive molecules in blood circulation [11, 12], such as LDL. Its oxidation triggers the formation of ox-LDL, resulting in tissue damage and cerebrovascular pathologies (e.g. atherosclerosis) [22, 50]. Here, we observed that WD increases ox-LDL levels in blood circulation of young and aged WD-fed animals in comparison to their ND-fed littermates. Noteworthy, these effects seem to be restricted to the periphery, as WD did not affect ox-LDL levels in the brain. Moreover, it has been demonstrated that blood ox-LDL interacts with cerebral endothelial cells *via* Lectin-like oxidized low-density lipoprotein receptor-1 (LOX-1), thus impairing cerebral microvasculature function [51, 52]. As such, ox-LDL, *via* endothelial LOX-1, induces the production and release of ROS (e.g. peroxynitrite (ONOO^−^)) by endothelial cells in the perivascular space and brain parenchyma (Figure 8) [51]. As a consequence of ROS production, it was reported that lipid peroxidation results in the formation of MDA, a highly reactive molecule that is one of the most prevalent lipid peroxidation products during oxidative stress (Figure 8) [33, 50]. In our study, WD increased MDA accumulation in cerebral microvasculature in both young and aged mice. Thus, our observation suggests that WD exacerbates oxidative stress specifically in cerebral microvasculature, which might cause its dysfunction as previously reported [48].

As an other factor involved in cerebral microvasculature dysfunction, Aβ accumulation within cerebral microvasculature leads to CAA development, occurring in more than 80% of AD patients [27]. Neuropathological studies of severe CAA cases have outlined reduced circumferences of cerebral microvessels, loss of vascular smooth muscle cells (e.g. pericytes), exacerbated inflammation in the perivascular space and the accumulation of oxidative stress markers, which are thought to contribute to Aβ-dependent cerebral microvasculature dysfunction [53, 54]. In addition, population-based studies reported a strong correlation between CAA and cognitive deficits [55, 56]. In this regard, an experimental study showed that Tg2576 mice lacking CD36, which is a receptor implicated in Aβ trafficking, displayed selectively reduced soluble Aβ 1-40 levels and CAA development, and preserved pericytes function [57]. Interestingly, marked cognitive improvements were reported, while no changes were observed in parenchymal Aβ plaque deposition [57], thus indicating that vascular Aβ accumulation on its own is sufficient to cause a cognitive decline. Indeed, in our study we showed that WD exacerbates age-related accumulation of soluble Aβ 1-40 in cerebral MVs of aged WD-fed mice, which could contribute to cerebral microvasculature dysfunction and ultimately, to cognitive decline (Figure 8) [57].

Finally, we examined the function of cerebral microvasculature. Noteworthy, WD regimen used here did not affect BBB physical properties, as no infiltration of immune cells, IgG extravasation and changes in tight junction protein expression levels were observed in brains of young and aged APPswe/PS1 mice. As mentioned, pericytes play an important role in BBB function since their impairment or depletion is associated to major BBB dysfunction [28]. In our context, we did not observe any changes in pericyte coverage in the hippocampus of young and aged animal groups, thus indicating that WD did not affect pericyte viability. However, activation of PDGFRβ, a key receptor involved in pericyte function [36, 37], was decreased following the WD, which does not necessarily affect BBB physical permeability at this level. In addition, pericyte dysfunction was accompanied with reduced ABCB1 protein levels in cerebral MVs, thus indicating that, at this level, BBB transport function begins to be compromised. Thus, we propose that WD might induce pericyte dysfunction, contributing to cerebral microvasculature dysfunction and ultimately to cognitive decline, as previously shown [25, 37]. Interestingly, an experimental study has demonstrated that pericyte loss in mice overexpressing Swedish human APP (APP^sw/0^/Pdgfr^+/−^) aggravates AD-related pathology mainly by increasing brain soluble Aβ levels, which in turn self-amplifies Aβ-induced pericyte loss, thus exacerbating cerebral microvascular damage and cognitive impairments [31]. In our study, we observed that soluble Aβ 1-40 reduced the metabolic activity of pericytes (i.e. HBVP) *in vitro*, which was significantly worsened by oxidative stress (i.e. MDA). Taken together these results confirm our previous *in vivo* observations, which outline the direct contribution of WD-induced vascular Aβ 1-40 accumulation and oxidative stress, in cerebral microvasculature dysfunction, and ultimately, in cognitive decline (Figure 8).

Our results are in line with several recent studies demonstrating that high fat diet exacerbates AD-like pathology in different AD mouse models [19]. For example, it has been shown that high fat diet-induced diabetes in 3xTg-AD leads to Aβ accumulation in the brain and cognitive deficits, thus indicating an interrelation between metabolic disorders and AD pathogenesis in a different transgenic AD mouse model [58]. Importantly, the effects of high fat diet were rapidly reversed by a single systemic injection of insulin [58]. Interestingly, insulin has been previously shown to inhibit Aβ-induced cell death of pericytes *in vitro* [59]. Taken together, it is conceivable to speculate that insulin might have rescued pericyte survival in high fat diet-fed 3xTg-AD mice. Therefore, our study along with these previous studies outline an important pathological link between high fat diet and cerebral microvasculature function *via* pericytes, a link that can be exploited to develop new therapeutic strategies that emphasis on enhancing pericyte survival, thus preserving cerebral microvasculature function.

Our study provides new insights regarding the synergistic role of age and high fat diet, two major risk factors associated to AD [25], in cerebral microvasculature dysfunction observed in AD. This study contributes to improve our understanding of the mechanisms that mediate cerebral microvasculature dysfunction, highlighting the crucial role of high fat diet in exacerbating age-induced vascular Aβ accumulation, oxidative stress and pericyte dysfunction, thus suggesting their contribution to loss of synaptic plasticity and ultimately, cognitive decline. Additional studies are warranted to explore the molecular and cellular mechanisms involved in triggering cerebral microvasculature dysfunction in AD. Importantly, we believe that new therapeutic approaches should take into consideration the implication of age and high fat diet on their efficacy, in order to better translate experimental findings into clinics.

## MATERIALS AND METHODS

### Experimental design

Animal experiments were performed according to the Canadian Council on Animal Care guidelines, as administered by the Laval University Animal Welfare Committee. All efforts were made to reduce the number of animals used and to avoid their suffering. Three and 12 months old APPswe/PS1 transgenic mice harboring the human presenilin I (A246E variant) and the chimeric mouse/human Aβ precursor protein (APP695swe) under the control of independent mouse prion protein (PrP) promoter elements [B6C3-Tg(APP695)3Dbo Tg(PSEN1)5Dbo/J] (Jackson ImmunoResearch Laboratories Inc.) were maintained in a C57BL/6J background. Mice were housed and acclimated to standard laboratory conditions (12-hour light/dark cycle / lights on at 7:00 AM and off at 7:00 PM) with free access to chow and water. Only males were used at the age of 3 and 12 months. Three months old (i.e. young) mice were used since at this age AD-related pathology is not developed in the mouse line. Twelve months old (i.e. aged) mice were used since at this age AD-related pathology is well developed and brain Aβ loading is very high in the mouse line. Animals were fed (i.e. free feeding) for 4 months (i.e. 120 days) with a high fat “Western diet” [38] (WD) containing 42% kcal from fat, including 0.2% total cholesterol, more than 60% of total fatty acids and 34% by weight of sucrose (High fat diet: TD.88137, Harlan Laboratories Inc., Teklad Global diets) or normal diet (ND) containing 13% kcal from fat (Control diet: TD.08485, Harlan Laboratories Inc., Teklad Global diets). At the end of the protocol, animals were at 7 (i.e. young) and 16 (i.e. aged) months of age (*n* = 10-12 per group)

### Tissue collection

For molecular analyses, mice were deeply anesthetized *via* an intraperitoneal injection of a mixture of ketamine hydrochloride/ xylazine (100/10 mg/kg) and then transcardially perfused with ice-cold 0.9% saline solution (NaCl, Sigma-Aldrich) by using a peristaltic pump, brains were removed and immediately frozen in dry ice prior to storage in −80°C for subsequent molecular analyses. For immunofluorescence and histochemical analyses, removed brains were post-fixed in 4% paraformaldehyde (PFA) solution (Electron Microscopy Sciences) (pH 7.4) for 48 hours (h) at 4°C and then immersed in a 4% PFA solution containing 20% sucrose (Sigma-Aldrich) overnight at 4°C. Fixed brains were frozen with dry ice/ethanol mixture, mounted on a microtome (Leica) and cut into 25 μm coronal sections. The collected slices were placed in tissue cryoprotectant solution containing 0.05 M sodium phosphate buffer (pH 7.3), 30% ethylene glycol and 20% glycerol (Sigma-Aldrich), and stored at −20°C.

### Immunofluorescence staining

Free-floating sections were washed with potassium phosphate-buffered saline (KPBS) (Sigma-Aldrich) (3x, 10 minutes) and then incubated for 20 minutes (min) in either a permeabilization/blocking solution containing 4% goat serum (Multicell), 1% bovine serum albumin (BSA) (Sigma-Aldrich) and 0.4% Triton X-100 (Sigma-Aldrich) in KPBS or 100% methanol (Sigma-Aldrich) for permeabilization (used for Desmin). Sections were incubated overnight at 4°C with different primary antibodies diluted in the permeabilization/blocking solution. The following primary antibodies were used; mouse anti-human Aβ monoclonal antibody (6E10) (1/1500; cat. num.: SIG-39320, Covance Inc.) and rabbit monoclonal anti-mouse Desmin (1/250; cat. num.: ab32362, Abcam). Afterwards, the sections were rinsed in KPBS (3x, 10 min), followed by 2 h incubation with Cy3-conjugated goat anti-mouse secondary antibody (1/1000; cat. num.: 115-165-003, Jackson ImmunoResearch Laboratories). Sections were incubated overnight under light protected vacuum to allow an optimal fixation of brain sections on slides. Then, sections were rinsed in KPBS (3x, 10 min), stained with 0.0002% 4′,6-diamidin-2-fenilindolo (DAPI) for 5 minutes, rinsed again in KPBS (3x, 10 min), mounted onto SuperFrost slides (Fisher Scientific), and coverslipped with antifade medium composed of 96 mM Tris-HCl, pH 8.0, 24% glycerol, 9.6% polyvinyl alcohol, and 2.5% diazabicyclooctane (Sigma-Aldrich). Epifluorescence images were taken using a Nikon C80i microscope equipped with both a motorized stage (Ludl) and a Microfire CCD color camera (Optronics). For stereological analyses, 6E10-positive Aβ plaques or Desmin-positive microvessels were quantified using a Wacom pen tablet and the StereoInvestigator software package (6.02.1; MicroBrightField) by a blinded investigator.

### Brain infiltration and immunoglobulin extravasation

Free-floating sections were washed with KPBS (3x, 10 min) and then incubated for 20 min in the permeabilization/blocking solution containing 4% goat serum, 1% BSA (Sigma-Aldrich), and 0.4% Triton X-100 (Sigma-Aldrich) in KPBS. For brain infiltration, sections were incubated overnight at 4°C with rabbit monoclonal anti-mouse CD45 (1/500; cat. num.: 553076, BD Bioscience) diluted in the permeabilization/blocking solution. Afterwards, the sections were rinsed in KPBS (3x, 10 min), followed by 2 h incubation with biotin-conjugated goat anti-mouse secondary antibody (1/1000; cat. num.: BA9200, Vector Laboratories). For immunoglobulin extravasation, sections were directly incubated for 2 h with biotin-conjugated goat anti-mouse secondary antibody (1/1000; cat. num.: BA9200, Vector Laboratories). The Biotin-conjugated secondary antibodies were detected using the avidin peroxidase kit (Vectastain ABC kit, Vector Laboratories) and diaminobenzidine (Sigma-Aldrich), following the manufacturer's instructions. Sections were then mounted onto SuperFrost slides (Fisher Scientific), dehydrated and coverslipped with DPX mounting medium (Electron Microscopy Sciences). Bright light images were taken using the Nikon C80i microscope equipped with the motorized stage (Ludl) and a Microfire CCD color camera (Optronics). For IgG extravasation, sections were digitized prior to densitometric analysis of IgG-positive coverage area (i.e. extravasation) using ImageJ software.

### Soluble Aβ 1-42/ and 1-40 ELISAs

Brain levels of soluble Aβ 1-42 and 1-40 were quantified using Human Amyloid β 1-42 and 1-40 Brain ELISA kits (EMD Millipore) respectively. The experimental procedure for Aβ 1-42 and 1-40 detection was performed according to the manufacturer's instructions. Absorbance was acquired using a microtiter plate reader (SpectraMax 340PC, Molecular Devices), and analyzed using SOFTmax Pro3.1.1 software (Molecular Devices).

### Cerebral microvessel isolation

Cerebral microvessels (MVs) were isolated on dextran gradient as described previously [60], with slight modification. Briefly, the cerebellum, meninges, brainstem and large superficial blood vessels were removed and the remaining cortices were gently homogenized in a Teflon glass homogenizer in ice-cold microvessels (i.e. capillaries) isolation buffer (MIB) containing 15 mM HEPES, 147 mM NaCl, 4 mM KCl, 3 mM CaCl2, and 12 mM MgCl2 (Sigma-Aldrich) supplemented with 5% Protease Inhibitor Cocktail (P8340; Sigma-Aldrich) and 1% Phosphatase Inhibitor Cocktail 2 (P5726; Sigma-Aldrich). Brain homogenates were centrifuged at 1000 xg for 10 min at 4°C. The resulting pellets were resuspended in 30% dextran (molecular weight, 64 to 76 kDa, Sigma-Aldrich) in MIB. Suspensions were centrifuged at 4400 xg for 20 min at 4°C. The resulting crude brain capillaries-rich pellets were resuspended in MIB and filtered through two nylon filters of 100- and 60-μm mesh size (EMD Millipore). The quality of trapped cerebral MVs in the final filtrates was checked with a bright-field microscopy. Filtrates were centrifuged at 1000 xg, and the resulting pure brain capillaries were stored at −80°C.

### Protein extraction

Isolated cerebral MVs or total brain tissues were homogenized and lysed in NP-40 lysis buffer supplemented with 5% Protease Inhibitor Cocktail and 1% Phosphatase Inhibitor Cocktail 2 (Sigma-Aldrich). Lysate samples were sonicated over two cycles lasting 20 s each at 4°C at 40% power (Sonic dismembrator, model 100, Fisher Scientific). Protein concentrations were measured by means of the Quantipro BCA assay kit (Sigma-Aldrich) according to the manufacturer's protocol. Absorbance was acquired using a microtiter plate reader (SpectraMax 340PC, Molecular Devices), and analysed using SOFTmax Pro3.1.1 software (Molecular Devices).

### Gelatinase activity assay

Matrix metalloproteinases (MMP)-2/9 enzymatic activities were measured by a fluorescent based assay “EnzCheck^®^ Gelatinase/ Collagenase Assay Kit” (Molecular Probes, Eugene). The gelatinase assays were performed accordingly to the manufacturer's protocol.

### Malondialdehyde quantification

Malondialdehyde (MDA) was quantified by a colorimetric assay “OxiSelect^®^ TBARS Assay Kit” (STA-330, Cell Biolabs). The TBARS assays were performed accordingly to the manufacturer's protocol.

### MCP-1 quantification

MCP-1 was quantified in plasma samples using the BD Cytometric Bead Assay (CBA) Mouse inflammation kit (552364, BD Bioscience). The CBA was performed accordingly to the manufacturer's protocol.

### Western blot analysis

For total and phosphorylated protein analyses, 2X SDS loading buffer was added to lysate samples containing equal amounts of protein (10 μg) and heated (95°C, 5 minutes), except for ABCB1, prior to protein analysis. Samples were subjected to SDS-polyacrylamide gel electrophoresis (SDS-PAGE) using 4-15% precast polyacrylamide gel (Bio Rad) followed by Western blot analysis, with primary antibodies diluted 1/1000 in 5% skim milk (Sigma-Aldrich) and 0.1 M tris-buffered saline-Tween X-100 (TBS-T). The following antibodies were used: rabbit anti-claudin-5 (1/1000; cat. num: sc-28670, Santa Cruz Biotechnology), rabbit anti-occludin (1/1000; cat. num.: 71-1500, Life Technologies Inc.) rabbit anti-PDGFRβ (1/1000; cat. num.: ab32570, Abcam), mouse anti-Phospho-tyrosine (1/1000; cat num.: 9411, Cell signaling), rabbit anti-ABCB1 (1/1000; cat. num: sc-8313, Santa Cruz), rabbit anti-BDNF (1/1000; cat. num.: ANT-010, Alomone Labs), anti-β-actin (1/40000; cat. num.: MAB1501; EMD Millipore) and anti-α-tubulin (1/40000; cat. num.: 625902, Biolegend). Primary antibodies were detected with horseradish peroxidase (HRP)-conjugated secondary antibody that were diluted 1/5000 in 5% skim milk and TBS-T and revealed by enhanced chemiluminescence plus (ECL) solution (GE Healthcare Life Sciences). Blots were digitized, densitometrically analyzed with ImageJ image analysis software (NIH), corrected for protein loading by means of β-actin or α-tubulin, and expressed as optical density (O.D.) values comparing ND with WD groups.

### Immunoprecipitation

Total brain homogenates (TBH), i.e. brain lysate samples; have been obtained as described in the protein extraction section. Lysates containing 500 μg of proteins were supplemented with sodium orthovanadate (Sigma-Aldrich) (final concentration: 1 mmol/L) and complemented with 3 equal volumes of NET buffer (100 mmol/L Tris, 200 mmol/L NaCl, 5 mmol/L EDTA, 5% NP-40, pH 7.4) [26]. Two micrograms of anti-PDGFRβ antibody (Abcam) were added to each sample and incubated overnight at 4°C under slight rotation. The next day 20 μL of Protein A/G plus-Agarose (Santa Cruz Biotechnology) was added to the samples, which were incubated 1 h at 4°C. Finally samples were centrifuged for 30 seconds (s) at 15 000 rpm at 4°C. Supernatants were dispersed and pellets washed 3 times in ice cold NET buffer. Twenty microliters of 2X SDS loading buffer was added to each pellet and boiled for 5 min prior to a short centrifugation at 4000 rpm in order to precipitate beads. Supernatants were then subjected to Western blot analysis for phospho-tyrosine (Cell signaling).

### Flow cytometry analysis

For monocytes, facial vein blood was collected in EDTA coated vials (Sarstedt). Flow cytometry analysis was performed as described [60]. Briefly, 50 μL of total blood was incubated on ice for 15 minutes with 4 μl purified rat anti-mouse CD16/CD32 antibody (BD Bioscience) diluted in 35 μL of DPBS. Always on ice, the mixture of cells and anti-mouse CD16/CD32 was incubated with AF700-conjugated anti-CD11b antibody (1/100, eBioscience), APC (allophycocyanin)-conjugated anti-CD115 antibody (1/100, eBioscience), V500-conjugated anti-CD45 antibody (1/100, BD BioScience), V450-conjugated anti-Ly6-C antibody (1/100, BD BioScience) and PE-conjugated anti-Ly6G antibody (1/100, eBioscience) for 45 minutes. Red blood cells were lysed with 1.5 mL of 1X Pharm Lyse buffer, accordingly to manufacturer's protocol (BD BioScience). After hemolysis, remaining cells were washed with DPBS and resuspended in equal volumes of DPBS. For hematopoietic stem cells (HSC), mice were anesthetized and transcardially perfused as above prior to isolation of the bone marrow. Bone marrow cells were collected by flushing the femurs of mice with ice cold DPBS + 5% fetal bovine serum (FBS, Sigma-Aldrich) by using 10 ml syringe with a 25G 1 ½ needle. Bone marrow cells were filtered on 35 μm nylon mesh (BD Bioscience) and washed three times in FBS-free DPBS (centrifugating at 300 xg for 5 min). Bone marrow cells were resuspended in FBS-free DPBS containing 4% of rat serum (Multicell) and incubated on ice for 30 min. Always on ice, bone marrow cell suspensions were incubated with Live/Dead-blue staining (1/50, Life technologies), V450-conjugated anti-Lineage cocktail (1/50, eBioscience), FITC-conjugated anti-CD34 antibody (1/20, eBioscience), AF700-conjugated anti-CD16/32 antibody (1/50, eBioscience), PE TexasRed-conjugated anti-CD117 (c-Kit) antibody (1/100, eBioscience), V500-conjugated anti-Sca-1 (Ly6A/E) antibody (1/50, BD BioScience) and APC (allophycocyanin)-conjugated anti-CD115 antibody (1/100, eBioscience) for 45 min. Finally, bone marrow cells were analyzed using a LSR II flow cytometer and data acquisition was done with BD FACS Diva software (Version 6.1.2, BD Bioscience). The results were analyzed using FlowJo software (Version 7.6.1, Tree Star Inc.)

### Quantitative real-time PCR

Tissus were homogenized in Qiazol buffer (Qiagen, Germantown, MD, USA) and total RNA was extracted using the RNeasy kit on-column DNase (Qiagen, Hilden, DE) treatment following the manufacturer' instructions. Quantity of total RNA was measured using a NanoDrop ND-1000 Spectrophotometer (NanoDrop Technologies, Wilmington, DE, USA) and total RNA quality was assayed on an Agilent BioAnalyzer 2100 (Agilent Technologies, Santa Clara, CA, USA). First-strand cDNA synthesis was accomplished using 5 ug of isolated RNA in a reaction containing 200 U of Superscript IV Rnase H-RT (Invitrogen Life Technologies, Burlington, ON, CA), 300 ng of oligo-dT18, 50 ng of random hexamers, 50 mM Tris-HCl pH 8.3, 75 mM KCl, 3 mM MgCl2, 500 uM deoxynucleotides triphosphate, 5 mM dithiothreitol, and 40 U of Protector RNase inhibitor (Roche Diagnostics, Indianapolis, IN, USA) in a final volume of 50 ul. Reaction was incubated at 25°C for 10 min, then at 50°C for 20 min and inactivated at 80°C for 10 min. PCR purification kit (Qiagen, Hilden, DE) was used to purify cDNA. Oligoprimer pairs were designed by GeneTool 2.0 software (Biotools Inc, Edmonton, AB, CA) and their specificity was verified by blast in the GenBank database. The synthesis was performed by IDT (Integrated DNA Technology, Coralville, IA, USA) (Table 1). cDNA corresponding to 20 ng of total RNA was used to perform fluorescent-based Realtime PCR quantification using the LightCycler 480 (Roche Diagnostics, Mannheim, DE). Reagent LightCycler 480 SYBRGreen I Master (Roche Diagnostics, Indianapolis, IN, USA) was used as described by the manufacturer. The conditions for PCR reactions were: 45 cycles, DMSO 2% denaturation at 95°C for 10 sec, annealing at 59°C for 10 sec, elongation and reading at 72°C for 14 sec. A melting curve was performed to assess non-specific signal. Relative quantity was calculated using fit point method and by applying the delta Ct method [61]. Normalization was performed using the reference genes shown to be genes having stable expression levels from embryonic life through adulthood in various tissues [62]: ATP synthase, H+ transporting, mitochondrial F1 complex, O subunit (*atp5o*), hypoxanthine guanine phosphoribosyl transferase 1 (*hprt1*) and glyceraldehyde-3-phosphate dehydrogenase (*gapdh*). Quantitative Real-Time PCR measurements were performed by the CHU de Québec Research Center (CHUL) Gene Expression Platform, Quebec, Canada and were compliant with MIQE guidelines [63].

**Table 1:** Sequence primers and gene description for RT-qPCR experiment

Gene Symbol	Description	GenBank	size (pb)	Primer sequence 5′→3′ S/AS
MMP9	Mus musculus matrix metallopeptidase 9 (Mmp9)	NM_013599	166	CAGCTGGCAGAGGCATACTTGTA/GTGGTGTTCGAATGGCCTTTAGT
TIMP1	Mus musculus tissue inhibitor of metalloproteinase 1 (Timp1), 3 transcripts	NM_001044384	137	TGTGGGAAATGCCGCAGATATC/TGATGTGCAAATTTCCGTTCCTTAG
BDNF	Mus musculus brain derived neurotrophic factor (Bdnf), 4 transcripts	NM_007540	130	CCCAACGAAGAAAACCATAAGGAC/TGTTTGCGGCATCCAGGTAATTTTTGT
NOS1	Mus musculus nitric oxide synthase 1, neuronal (Nos1)	NM_008712	129	AAGCCCTGGTGGAGATTAACATTG/CCCCTCTGCAGCGGTACTCAT
PLAT	Mus musculus plasminogen activator, tissue (Plat)	NM_008872	194	CGGCCTGGGCAGACACAATTAT/AAGGGTGTGAGGTGATGTCTGTGTAG
Atp5o	Mus musculus ATP synthase, H+ transporting, mitochondrial F1 complex, O subunit	NM_138597	142	GCTATGCAACCGCCCTGTACTCTG/ACGGTGCGCTTGATGTAGGGATTC
Hprt1	Mus musculus hypoxanthine guanine phosphoribosyl transferase 1	NM_013556	106	CAGGACTGAAAGACTTGCTCGAGAT/ CAGCAGGTCAGCAAAGAACTTATAGC
GAPDH	Mus musculus glyceraldehyde-3-phosphate dehydrogenase	NM_008084	194	GGCTGCCCAGAACATCATCCCT/ ATGCCTGCTTCACCACCTTCTTG
ADNg Ctrl	Mus musculus chromosome 3 genomic contig, strain C57BL/6J (HSD3B1 intron)	NT_039239	209	CACCCCTTAAGAGACCCATGTT/ CCCTGCAGAGACCTTAGAAAAC

### Neurobehavioral tests

#### T-water maze

The T-water maze paradigm, a left/right discrimination test that assess the hippocampal-based learning and retention of mice, was performed in the weeks following the diet [64]. The T-maze apparatus (length of stem, 64 cm; length of arms, 30 cm; width, 12 cm; height of walls, 16 cm) was made of clear fiberglass and filled with water (23 ± 1°C) at a height of 12 cm. An escape platform (11 × 11 cm) was placed at the end of the target arm and was submerged 1 cm below the surface. The position of the platform was chosen randomly for each animal prior to testing. In the acquisition-learning phase, the mice were placed in the stem of the T-maze and swam freely until they found the submerged platform (located either in the right or in the left arm of the T-maze apparatus) and escaped to it. After reaching the platform, the mice remained on it for 20 s and then placed back in the maze for up to a maximum of 24 trials, except for a 10 minutes rest period after each 10 trial block. All trials were performed on one single day. A mouse was considered to have achieved criterion after 5 consecutive errorless trials. The reversal-learning phase was then conducted 2 days later, with the protocol repeated except that the mice were trained to find the escape platform on the opposite side. During the acquisition/reversal phase the platform was located in the same position for the entire stage. The number of trials to reach the criterion (5 of 5 correct choices made on consecutive trials) and the average of swimming speeds were recorded and analyzed.

### New object recognition

The New object recognition (NOR) paradigm, assessing the learning and memory of mice based on their innate preference for novelty [65], was performed in the weeks following the diet. The NOR is performed in a rectangular open field with the same size as their home cage (width: 18 cm, length: 28 cm, height: 12 cm) made of clear plexiglas. During training phase, mice were placed with two identical objects and allowed to explore both for 5 min. Animals whose exploration time was lower than 10 s per item were considered insufficient and not used for analysis. After 3 h, retention test was performed. Mice were left for 5 min in the experimental arena in the presence of a familiar object along with a new one comparable in size, texture and shape. For one-half of the animal, the new object was presented at the right side and at the left side for the other half. A digital camera was mounted on the ceiling above the arena and connected to a computer with a video-tracking system (ANY-maze, Stoelting Co), which objectively monitored and quantified movements. Object exploration was defined as touching the object or directing the nose toward it at less than 2 cm. The recognition index (NOR-index) represents the time spent exploring the novel object per total time.

### *In vitro* experiments

The human brain vascular pericyte (HBVP) cell line were cultured at 37°C in 5% CO2, 95% air in Pericyte medium (PM) (ScienceCell Inc.) containing 4% FBS, 1% Pericyte growth supplement (PGS), 100 U/mL penicillin and 100 μg/mL streptomycin (ScienceCell Inc.). HBVP are characterized by using antibody specific to | |-smooth muscle actin and are negative for HIV-1, HBV, HCV, mycoplasma, bacteria, yeast, and fungi. In all experiments, cells were grown to 70-90% confluence and subjected to a maximum of 20 cell passages. For the metabolic activity assay, 1×10^4^ HBVP were incubated with 0.5 μg of human soluble Aβ 1-40 monomers (cat. num.: AS-24236-5, AnaSpec) and/or 1 mM of malondialdehyde tetrabutylammonium salt (MDA; cat. num.: 63287, Sigma Aldrich) for 24 h prior to the absorbance based “XTT Cell viability” assay kit (cat. num.: 9095, Cell Signaling). The cell viability assays were performed accordingly to the manufacturer's protocol.

### Statistical analysis

Results are expressed as mean ± standard error of the mean (SEM). For the cellular and molecular analysis, the comparison between the group data was analyzed using standard two-tailed unpaired *t*-test or two-way ANOVA (sources of variation: age and diet, population (n) size: 10-12 animals) followed by Bonferroni's post-hoc tests. For neurobehavioral analyses, the comparison between the group data was analyzed using two-tailed paired *t*-test (for new object recognition test) or two-way ANOVA (sources of variation: age and diet, population (n) size: 10-12 animals) followed by Bonferroni's post-hoc tests. Correlation analyses were performed using two-tailed nonparametric correlation (spearman) test. A *p* value *<* 0.05 was considered statistically significant (95% confidence interval). All analyses were performed using GraphPad Prism Version 5.0 for Mac OS X (GraphPad Software).

### Image preparation

All the panels presented were assembled using Adobe Photoshop CS5 (version 12.0.4) and Adobe Illustrator CS5 (version 15.0.2).
